# LärMiljö (Learning Environment) - study protocol: movement, outdoor learning and well-being in school: Nina Simonsen

**DOI:** 10.1093/eurpub/ckac131.337

**Published:** 2022-10-25

**Authors:** N Simonsen, N Wackström, J Ray

**Affiliations:** 1 Folkhälsan Research Center, Public Health Research Program, Helsinki, Finland; 2 University of Helsinki, Department of Public Health, Helsinki, Finland

## Abstract

**Background:**

Outdoor education (OE) is a teaching method aiming to promote children’s learning, physical activity (PA) and wellbeing. OE in green areas may further increase positive effects. There is little knowledge on use of OE and its possible effects in a Finnish context. Also, there is a need to evaluate OE from a teacher perspective. The aim of the LärMiljö-study is to: a) survey the use of OE in Swedish-language primary schools and investigate factors related to its use; b) investigate the associations between OE and PA, wellbeing, nature relations and learning among children aged 9-13 years, considering other related factors.

**Methods:**

The self-determination theory (SDT) is used as theoretical framework. Data is collected via electronic surveys in Swedish-language primary schools, including: a) national surveys among principals and teachers; b) surveys among children and guardians. Children’s PA is measured for a 7-day period via accelerometers and a diary is kept. Teachers keep a class diary on OE provided. Academic tests are performed. Data will be analyzed using quantitative methods.

**Results:**

This abstract describes the study protocol. The investigation of associations between OE and outcomes among children is mainly done by comparing classes that regularly use OE with classes that do not. Main outcomes are PA, psychosocial wellbeing, academic performance and nature connectedness; secondary outcomes are school motivation, social relations and sleep. Teachers’ experience and use of OE is explored, as is perceived effects of and barriers to OE use. Based on SDT, associations between OE and need satisfaction at work, competence, motivation and work engagement are studied.

**Conclusions:**

The study represents a unique opportunity to explore OE use in primary schools, its effects among children and teachers, and what the supporting and hindering factors for its use are. The study contributes knowledge that can be used to promote learning and wellbeing in school.

**Key messages:**

• Outdoor education in primary school may promote schoolchildren’s learning, physical activity, wellbeing and nature relation, but more knowledge is needed, including the perspective of teachers.

• The LärMiljö-study will broaden the understanding of the potential effects of OE in primary education and school health promotion, and on factors supporting and hindering its use.

